# High-throughput RNA interference screening using pooled shRNA libraries and next generation sequencing

**DOI:** 10.1186/gb-2011-12-10-r104

**Published:** 2011-10-21

**Authors:** David Sims, Ana M Mendes-Pereira, Jessica Frankum, Darren Burgess, Maria-Antonietta Cerone, Cristina Lombardelli, Costas Mitsopoulos, Jarle Hakas, Nirupa Murugaesu, Clare M Isacke, Kerry Fenwick, Ioannis Assiotis, Iwanka Kozarewa, Marketa Zvelebil, Alan Ashworth, Christopher J Lord

**Affiliations:** 1 The Breakthrough Breast Cancer Research Centre, The Institute of Cancer Research, 237 Fulham Road, London, SW3 6JB, UK

## Abstract

RNA interference (RNAi) screening is a state-of-the-art technology that enables the dissection of biological processes and disease-related phenotypes. The commercial availability of genome-wide, short hairpin RNA (shRNA) libraries has fueled interest in this area but the generation and analysis of these complex data remain a challenge. Here, we describe complete experimental protocols and novel open source computational methodologies, shALIGN and shRNAseq, that allow RNAi screens to be rapidly deconvoluted using next generation sequencing. Our computational pipeline offers efficient screen analysis and the flexibility and scalability to quickly incorporate future developments in shRNA library technology.

## Background

RNA interference (RNAi) facilitates the assessment of gene function by silencing gene expression using synthetic anti-sense oligonucleotides or plasmids. It exploits a physiological mechanism that represses gene expression, primarily by causing the degradation of mRNA transcripts. In mammalian cells, physiological RNAi is primarily mediated by non-protein-coding RNA transcripts, known as microRNAs (miRNAs). miRNAs are produced in a similar manner to mRNAs, but miRNAs are processed into shorter RNA species containing a hairpin structure, known as short-hairpin RNAs (shRNAs). shRNAs are in turn processed into short double-stranded pieces of RNA known as short interfering RNAs (siRNAs). Within the multi-protein RNA-induced silencing complex (RISC), one strand of a siRNA duplex binds a protein-coding mRNA transcript that bears a complementary nucleotide sequence. This interaction allows a nuclease in the RISC to cleave and destroy the protein-coding mRNA, therefore silencing the expression of the gene in a relatively sequence-specific manner.

The experimental use of synthetic siRNAs and shRNA-expressing plasmids has profoundly changed the way in which loss of function experiments can be performed. Previously, techniques that were either more time consuming (gene targeting), or capricious (antisense RNA), were used. Now libraries of RNAi reagents can be purchased and used to silence almost any gene at will. While siRNAs are typically used in multiwell plate-based screening, shRNAs are commonly used for pooled competitive screening approaches, often called barcode screening.

Barcode screening offers improvements in speed and scale compared to plate-based screening. In barcode screening, a large population of cells is infected or transfected with a pool of different shRNA vectors. Cells are then split into two groups and one group is treated differently from the other - for example, with a drug. After this selective pressure is applied, cells are harvested from both populations and integrated hairpins extracted from the genomic DNA of each population by PCR. The relative quantity of each hairpin in the two populations is then compared, to identify those genes that modulate the response to the perturbation in question. For example, in the case of drug screens, hairpins that are over- or under-represented in the drug treated sample compared to the control sample could be considered as targeting genes that modulate sensitivity or resistance to the drug, respectively.

Traditionally, Sanger sequencing has been used as a readout for positive selection screens. However, this approach is costly, time consuming and in general not scalable. In the case of negative selection screens, microarray hybridization is frequently used as a readout [1,2]. This approach requires the production of custom microarray chips for each library, has a limited dynamic range and is restricted by the varying effectiveness of individual probes. Next generation sequencing (NGS) technologies have recently emerged as a cost-effective means of generating large quantities of sequence data in a short time. Using massively parallel sequencing in place of Sanger sequencing or microarray-based approaches offers several potential advantages in terms of flexibility of input library, scalability and dynamic range.

Already, a small number of laboratories have used shRNA screens coupled to NGS [1,3-5]. One critical issue that limits the wider exploitation of this technology is the absence of a freely available and simple package for the analysis of shRNA NGS data. With this in mind, we describe here detailed protocols for pooled shRNA screening coupled to NGS screen deconvolution. As part of our optimization of this technology, we have also developed a computational pipeline to analyze NGS data from shRNA screens and describe two open source analysis packages, shALIGN and shRNAseq, designed to simplify barcode screen analysis. Using shRNA pools with engineered depletion, we also assess the sensitivity and reproducibility of this method. As the cost of both shRNA libraries and NGS is rapidly decreasing, these methods and analytical tools may aid the wider adoption of this powerful technology.

## Results and discussion

**Figure 1:**
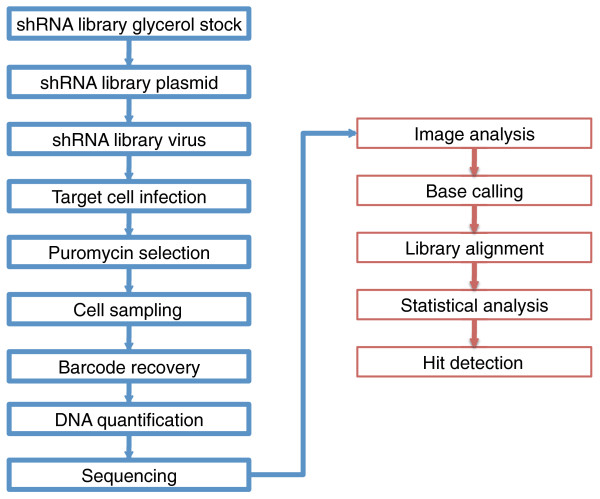
**Workflow of a typical shRNA barcode screen**. The steps in blue boxes represent the experimental phase, whereas the steps in red boxes represent the computational analysis phase.

shRNA barcode screening is a lengthy procedure that required considerable optimization. Here we describe how methods were selected from principles and procedures established by McManus [3], Hannon, Elledge and Lowe [2,4,5] and optimized for the entire shRNA barcode screening workflow from library production to statistical analysis (Figure 1).

### Bacterial culture

**Figure 2:**
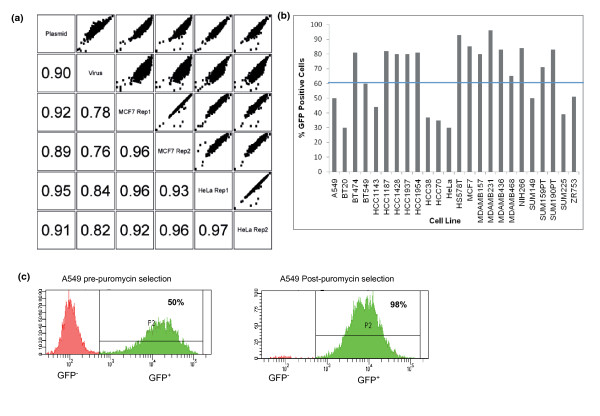
**GIPZ library plasmid/viral pool production and target cell line infection**. **(a) **Scatter plots showing pair-wise comparisons of log2 normalized read counts from shRNA plasmid, virus and two technical replicates of shRNA constructs amplified from genomic DNA 3 days post-infection of MCF7 and HeLa cells. Numbers indicate Pearson correlation between conditions. Technical replicates show high correlation. Plasmid shows high correlation with infected cells in both cell lines. Virus shows weaker correlation with both plasmid and infected cells. **(b) **Test infection of a panel of breast cancer cell lines. Most cell lines show >60% green fluorescent protein (GFP)-positive cells 3 days after infection. Those that did not were puromycin selected to increase the population of GFP-positive cells to >90%. **(c) **Fluorescence-activated cell sorting (FACS) profiles showing the percentage of GFP-positive cells before and after puromycin selection in A549 cells.

One factor that could affect screen performance is the variation of representation of individual hairpins within a screening pool. Since library production relies on the growth of thousands of bacterial cultures, it is inevitable that there will be some variation in growth in individual wells within a plate, and between plates within a screening pool. Consequently, it is important to be systematic about the generation and pooling of bacterial cultures. First, all liquid handling was performed robotically to ensure that most errors are systematic and can be easily traced. Second, growth temperatures and times were tightly controlled. Culture plates were stacked evenly to ensure even air circulation to all plates and wells. Hairpin plasmids were grown in small batches (ten plates) to facilitate quality control. Since recombination was a problem in previous generations of shRNA libraries, the quality of plasmid DNA was checked by restriction enzyme digest following plasmid purification. Once screening pools had been constructed, the plasmid pool was sequenced on the Illumina Genome Analyzer IIx (GAIIx) to determine hairpin representation (Figure 2a), using a shRNA targeted sequencing procedure based on that described by Zuber [4], Silva [5] and colleagues. Although it is somewhat difficult to normalize the representation of individual hairpins in large screening pools, it is important to minimize the variation within the population to reduce the chances that observed screen results can be attributed to issues in starting hairpin abundance. Although these issues can be partially mitigated at the statistical analysis stage (see below), careful library preparation and quality control can minimize variance in shRNA representation.

### Lentiviral packaging

Packaging of hairpin plasmid into lentiviral vectors requires large numbers of packaging cells and high transfection efficiency to ensure faithful representation of the plasmid pool in the viral supernatant. We have successfully employed two approaches to transfection of shRNA plasmids into packaging cell lines, calcium phosphate- and lipid-based transfection. Both methods were routinely used and returned viral supernatants of similar titer (data not shown). cDNA generated from viral supernatant was sequenced and compared to the plasmid DNA to ensure good representation of the library has been achieved (Figure 2a). Typically, cDNA from viral supernatant showed slightly greater variance in hairpin representation than the plasmid pool. Furthermore, hairpin representation at early time points post-viral integration demonstrated better correlation with plasmid representation than with virus (Figure 2a). This suggested that the viral cDNA preparation step was a considerable source of noise and thus plasmid shRNA sequence most likely represents a better reference for starting hairpin representation than virus. This analysis also demonstrated a high concordance between technical replicates, where the same DNA library was sequenced on different GAIIx runs.

Typically, lentiviral stocks were transduced using a multiplicity of infection (MOI) of 0.7 to reduce the likelihood of multiple integrations per cell and the emergence of combinatorial phenotypes. Accurate determination of viral titer in target cell lines allowed subsequent infection of screening cell lines at intended efficiencies. We tested a wide range of breast tumor cell line models and the majority infected at >60% using viral titers of 10^6 ^to 10^7 ^TU/ml (Figure 2b). Those that did not infect at high efficiency were puromycin selected to give a final green fluorescent protein (GFP)-positive cell population of >90% (Figure 2c).

### Viral transduction and cell sampling

Regardless of the design of a particular screen, the manner in which the viral transduction and subsequent cell culture are performed is crucial to the success of the screen. The maintenance of hairpin representation (the number of cells infected with each shRNA) and logarithmic cell growth are of particular importance [3-5]. Throughout all shRNA barcode screens, we maintained an average representation of 1,000 cells per shRNA construct to maximize the potential for phenotypic effects from each shRNA being observed in the final analysis. Previous screens have shown similar levels of shRNA representation to be suitable for the detection of shRNA depletion and enrichment [3,4]. Since barcode screening is a competitive growth screen, ensuring cells are in log growth at all times during the screen is critical to minimize changes in representation caused by localized restriction of cell growth due to over-confluence. Consequently, we recommend ensuring that cells are never allowed to achieve more than 70% confluence.

After viral integration and puromycin selection were complete, cultures were divided into two or more sets, depending on the experimental design. For example, in a typical drug sensitivity/resistance screen, cultures are divided into reference (vehicle treated) and test (drug treated) sets. Alternatively, in a simple viability screen, a sample of cells can be taken and stored for analysis at each passage, to generate a viability time-course. One of the strengths of the lentiviral system is the stable integration of hairpins; this allows the use of longer experimental time-courses than could generally be performed using siRNA screening. As a consequence, final screen results were typically assessed 2 to 3 weeks after dividing the cells into two arms. Every time the cultures were divided or sampled, aliquots were taken to assess the cell number (to construct growth curves) and the percentage of GFP-positive cells (to assess the number of cells required to maintain hairpin representation). To minimize screen variability, we use the same passage cells for each screen replicate. We also maintain consistent batches of media, serum, viral supernatant and tissue culture plasticware for all screen replicates, again to minimize experimental variation.

### Barcode recovery

**Figure 3:**
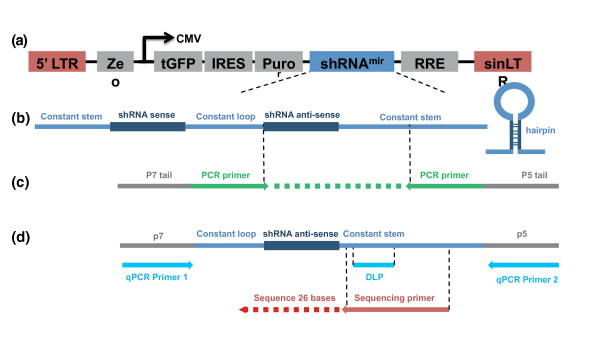
**PCR amplification, quantitative PCR and Illumina sequencing schema**. **(a) **Diagrammatic representation of the complete integrated shRNA construct. LTR, long terminal repeat; Ze, zeomycin resistance bacterial selectable marker; tGFP, turbo GFP; IRES, internal ribosome entry site; Puro, puromycin mammalian selectable marker; RRE, Rev response element; sinLT, self-interacting LTR. **(b) **The structure of the shRNAmir construct. The sense and antisense shRNA sequences hybridize to form a hairpin loop structure. **(c) **PCR primer alignment to the shRNA construct. The PCR primers incorporate p7 and p5 sequences to enable capture on an Illumina flowcell. **(d) **Sequencing primer, quantitative PCR (qPCR) primer and qPCR dual label probe alignment to the shRNA PCR product. CMV, cytomegalovirus; DLP, dual-labeled probe.

We used next generation sequencing to identify the frequency of each shRNA construct in screen cell populations. To facilitate this we used PCR amplification of genomic DNA from screen cell populations, based upon methods previously described by McManus [3], Hannon, Elledge and Lowe [2,4,5] and Quail [6]. PCR primers complementary to constant regions found in all shRNA constructs (Figure 3) were used to amplify the shRNA target sequence that is specific to each individual shRNA construct. The PCR primers also encompassed p5 and p7 sequences that allow sequence capture and sequencing-by-synthesis on the Illumina GAIIx platform.

To enable sufficient representation of each shRNA in the screening pool, multiple PCR reactions were performed in parallel to generate the sequencing library from each shRNA pool. For example, to maintain a representation of 1,000 cells per shRNA in a pool of 10,000 shRNAs at an MOI of 0.7, PCR amplification from 1 × 10^7 ^cells is required. Since a diploid human cell contains approximately 6 pg genomic DNA, we performed PCR amplification from 60 μg total genomic DNA using 30 parallel PCR reactions, each with 2 μg of genomic DNA.

### DNA quantification for next generation sequencing

**Figure 4:**
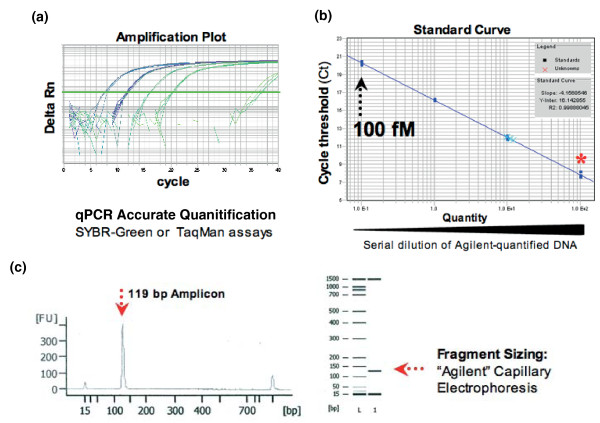
**Quantitative PCR quantification of PCR products**. Quantitative PCR (qPCR) assay designed to detect and quantify all amplifiable solexa molecules (using oligos p5/p7 and SybrGreen) or shRNA-specific PCR products (using Taqman, amplification primers p5/p7 and a dual-labeled probe). **(a) **shRNA PCR products quantified against a library of known concentration. **(b) **Standard curve constructed using a ten-fold dilution series covering 100, 10, 1 and 0.1 pM. **(c) **Agilent electrophoresis profile of reference library.

Accurate quantification of purified PCR products is required to achieve optimal cluster densities (the density of template clusters on the flowcell surface) in Illumina sequencing. Insufficient or excessive template results in poor sequencing yield with either scarce or overlapping signals. Real-time PCR has previously been used in Illumina sample preparation protocols to overcome the detection limits of capillary electrophoresis (typically 10 to 0.1 ng/μl) and enable standardization of cluster number per tile [6]. This led us to develop two robust quantification assays for Illumina DNA libraries (Figure 4).

First, we utilized the complement of the Illumina adapter sequences (p5 and p7, common to all Illumina sequencing libraries) as amplification primers in an intercalating dye-based quantitative PCR (qPCR) assay (SybrGreen). This is similar to an approach applied to the quantification of 454 Roche pyro-sequencing samples [7]. Second, for the quantification of DNA containing hairpin inserts in shRNA-derived Illumina libraries, we established a second strategy that utilized a dual-labeled probe (DLP) hydrolysis qPCR assay (Taqman). Here we designed a DLP complementary to a constant internal region in shRNA-specific Solexa PCR products. We based this approach on a reported alternative Taqman assay defined for an established Illumina sequencing application, pair-end RNA sequencing (RNAseq) [6]. Overall, the implementation of both of these innovative strategies allowed us to reliably quantify shRNA templates prior to massively parallel sequencing, leading to high numbers of mapped reads passing quality filters.

### Sequencing data analysis

**Figure 5:**
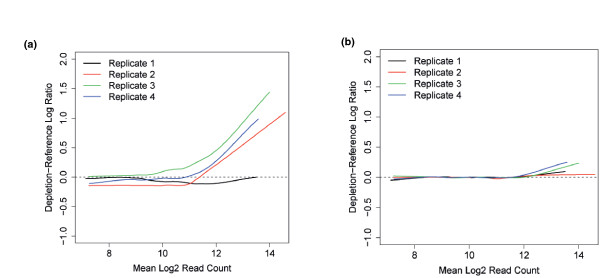
**Processing screen data to remove biases associated with differential hairpin abundance**. Plotting of the log ratio of paired samples (for example, reference-depletion) frequently revealed biases with respect to average hairpin abundance. Consequently, the data were normalized using loess regression to remove this bias. **(a) **The loess fit lines from four biological replicates of a 10k pool viability screen in MCF7 cells when the log ratio is plotted against log mean hairpin abundance. **(b) **The same plot post-loess normalization showing the standardization of the curves.

Raw images from the Illumina GAIIx were processed using the GApipeline (version 1.4 or higher) and resulting quality filtered short reads were aligned to the reference shRNA library using the shALIGN script. We developed shALIGN to circumvent a number of issues associated with other open source aligners commonly used in NGS analysis. Aligners such as Bowtie [8] and BWA (Burrows-Wheeler Aligner) [9] are specifically designed to align short reads to large genome sequences, rather than short shRNA sequences. Aligning reads to the genome would cause complications in the downstream analysis. First, using a whole genome alignment, reads could align to multiple genomic regions, which could allow the same read to be counted multiple times, or assigned at random to a particular location. Second, when using a whole genome alignment, reads with PCR or sequencing errors could align in different regions to unaltered reads, again giving a false picture of the number of reads mapping to a particular target. In contrast, shALIGN aligns reads directly to the target shRNA library, aligns each read to a single library construct, and ensures that all ambiguous reads are excluded from the final analysis. Typically, >90% of short reads aligned to the reference shRNA sequence library using this method. Resulting read counts per hairpin were statistically analyzed using a bespoke R-based package, shRNASeq. This analysis revealed systematic pool-specific biases in the log ratio of read counts from different screen arms at different levels of read abundance (Figure 5a). As a consequence, the log ratio was normalized to the average hairpin abundance using loess regression, and the normalized scores were re-scaled by the pool median absolute deviation (MAD; a robust estimator of variance) to ensure comparable distributions (Figure 5b). In some cases, we observed considerable heterogeneity in the distribution of normalized scores from biological replicates from the same screen, prompting us to perform a rank normalization across replicates to ensure identical distributions.

### Screen performance

**Figure 6:**
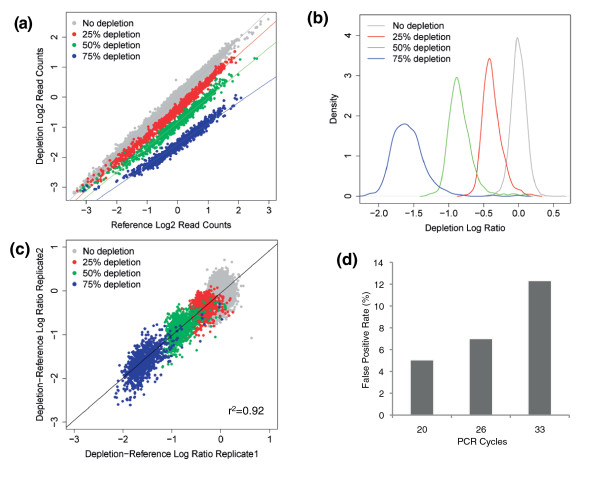
**Assessing the sensitivity and reproducibility of the screening platform**. We systematically depleted subsets of hairpins by 25%, 50% or 75% within a 10k pool and compared them to a non-depleted reference set 3 days after infection of MCF7 cells (see also Additional File 1). **(a) **Scatter plot of log2 normalized read counts from reference and depletion sets. **(b) **Density plot showing the distributions of the depleted hairpin subsets: 25% depleted hairpins are plotted in red, 50% depleted in green and 75% depleted in blue. The screening methodology was capable of detecting 50% depletion in hairpin representation with high accuracy in a single experiment. **(c) **Scatter plot of the depletion-reference log ratio from two biological replicates, indicating a high correlation (r2 = 0.92) and thus a reproducible screening method. **(d) **Plot depicting the false positive rate at a fixed false negative rate of 5% in a reference depletion experiment using different numbers of PCR cycles, indicating a decrease in the false positive rate with decreased PCR cycles.

We performed a number of experiments to evaluate screen performance in terms of sensitivity and reproducibility. To establish the sensitivity of the screening system, we performed a series of engineered depletion experiments (Additional file 1). To do this, we manually altered the representation of subsets of hairpins in a single screening pool, then performed a short-term screen (long enough for viral integration, but minimizing hairpin viability effects) and examined the difference in hairpin representation between the non-manipulated reference set and the systematically depleted set. These experiments demonstrated that we could detect 75% or 50% depletion in hairpin abundance with high accuracy in a single biological replicate (Figure 6a,b). Indeed, for the 75% depleted shRNA set, only seven depleted hairpins (0.74% of total depleted) could not be distinguished from the main population of non-manipulated hairpins at a threshold that detected no false positives (Table 1). The false negative rate was slightly increased in the 50% depleted hairpin set. Even when the shRNA constructs were depleted by 25%, half could still be distinguished from the main hairpin population with a 0% false positive rate (Table 1). The majority of false negatives in the 50% and 75% depleted hairpin groups were in hairpins with low starting representation. Filtering of the data to remove hairpins with low representation in the reference set resulted in a reduced false negative rate associated with a 0% false positive rate (Table 1). This appears to represent a considerable improvement in sensitivity in comparison with microarray-based methods. Previous work assessing the use of custom designed microarray sets to deconvolute shRNA screens showed that even the best-performing microarrays (barcode tiling arrays) gave a mean test/reference ratio of 0.78 for a 50% depletion [10], whereas our method offers a ratio of 0.54. Furthermore, barcode tiling arrays detected an 80% depletion with a test/reference ratio of 0.49 [10], whereas our NGS method gave a ratio of 0.33 for a depletion of 75%. Thus, the NGS-based approach provides a greater range of measurements and is better able to discriminate true depletions from background noise.

**Table 1:** Detection of depleted hairpins in reference depletion screens

	Un-filtered		Filtered	
	
Depletion	Min FPR	FNR	Min FPR	FNR
25%	0.00%	50.05%	0.00%	49.95%
50%	0.00%	4.21%	0.00%	2.89%
75%	0.00%	0.74%	0.00%	0.00%

The false positive and false negative rates at a range of log ratio thresholds were calculated by comparing each depletion group to the non-depleted hairpins. This was repeated for all hairpins and after filtering to exclude hairpins with low representation (<100 raw reads) in the reference set. The minimum false positive rate (Min FPR) found is shown along with the associated false negative rate (FNR).

Although NGS performs better than microarray hybridization for screen deconvolution, the barcode screening format is subject to a significant degree of stochastic noise regardless of the hairpin frequency detection method, and biological replication of screens is almost certainly required to overcome this. We performed two biological replicates of the reference depletion screen. There was a good correlation between log normalized read counts from replicate screens (reference arm r2 = 0.89). Similarly, a high correlation of depletion-reference log ratios was observed between biological replicates (r2 = 0.92; Figure 6c), suggesting that screens are highly reproducible. As expected, higher levels of noise were observed between biological replicates than between technical replicates.

Using the reference-depletion experimental framework, we also titrated the number of PCR cycles required to give faithful representation of the starting material. This demonstrated that PCR amplification acted as a source of noise in the experiment and that reducing the number of PCR cycles resulted in a decreased false positive rate at a set false negative rate of 5% (Figure 6d). Consequently, we opted to use 26 PCR cycles as this generated a visible band on the gel but still resulted in a relatively low error rate.

We also made use of the reference depletion dataset to assess the minimum amount of sequencing reads required to detect a reduction in shRNA representation. Many of the more novel sequencing platforms, such as the Illumina MiSEQ, are able to generate five million reads in a single run, offering the potential to use these cheaper platforms for shRNA screen deconvolution. We sampled reads from one reference depletion experiment to generate two additional datasets that contained either approximately 5 million reads or approximately 2.5 million reads, rather than the 10 million reads used in the previous analysis. This analysis revealed that a 50% reduction in shRNA representation could be detected with high sensitivity and specificity even in 2.5 million reads (Table 2; Additional file 2).

**Table 2:** Comparison of reference-depletion screens at different read depths

Total reads	Depletion	Min FPR	FNR
10 million	25%	0.00%	49.95%
10 million	50%	0.00%	2.89%
10 million	75%	0.00%	0.00%
5 million	25%	3.41%	42.35%
5 million	50%	0.02%	1.28%
5 million	75%	0.00%	0.00%
2.5 million	25%	9.65%	49.95%
2.5 million	50%	1.70%	1.07%
2.5 million	75%	0.00%	0.00%

The false positive and false negative rates at a range of log ratio thresholds were calculated by comparing each depletion group to the non-depleted hairpins after filtering to exclude hairpins with low representation (<100 raw reads) in the reference set. The minimum false positive rate (Min FPR) found is shown along with the associated false negative rate (FNR). We were able to consistently detect a 50% depletion in hairpin representation using a total of only approximately 2.5 million reads.

To test sensitivity in a genuine screen setting using different screening pool sizes, we performed a series of screens for viability in MCF7 cells. To assess screen performance, we used a number of genes where previous observations had shown that siRNA silencing inhibited MCF7 cells as measured using a viability assay based on cellular ATP levels [11]. We validated the viability effects of a set of GIPZ library shRNAs targeting these genes using single hairpin GFP competition studies. We also validated a number of negative control non-targeting shRNAs using this method. This revealed a total of six shRNAs, which caused a >50% depletion in GFP-positive cells over 2 weeks, along with 11 non-targeting hairpins that showed no viability phenotype in the same period (Additional file 3).

**Figure 7:**
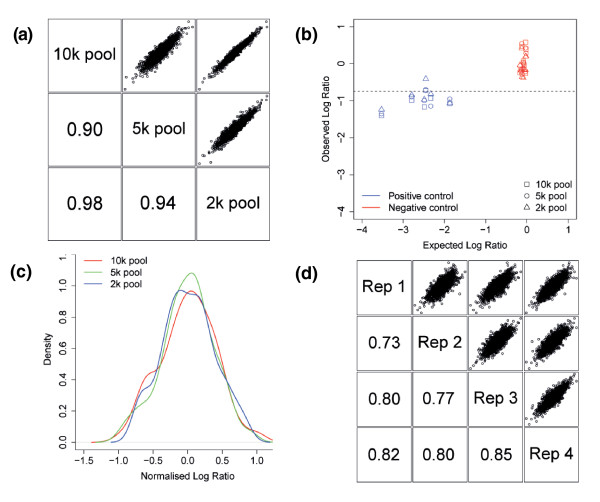
**MCF7 viability screen performance in different pool sizes**. **(a) **Scatter plots of 2,000 hairpins common to the 2,000, 5,000 and 10,000 shRNA pools showing high correlation of normalized scores (median of four replicates) between different pool sizes. Numbers indicate Pearson correlation between pools. **(b) **Plot of observed barcode screen log ratios for validated positive and negative controls in the 2,000, 5,000 and 10,000 shRNA pools versus expected scores based on single hairpin GFP competition assay scores. Positive controls are in blue and negative controls are in red. The horizontal dotted line indicates the threshold used for hit calling in the screen. Based on this threshold, 5 out of 6 valid positive controls were called hits whereas 0 out of 11 negative controls did not score as hits. **(c) **Distribution of log ratios of 101 non-targeting hairpins in the 2k pool. **(d) **Scatter plots of z-scores from four biological replicates of the 10k pool MCF7 viability screen, indicating a good agreement between replicates. Numbers indicate Pearson correlation between replicates.

Increased pool size leads to fewer reads per shRNA, and reduces screen sensitivity and potentially reliability. Therefore, we decided to compare the performance of pools containing 10,000, 5,000 or 2,000 shRNA constructs in a 14 day cell viability screen. To facilitate screen comparison, the 2,000 shRNA pool was a subset of the 5,000 shRNA pool and the 5,000 shRNA pool was a subset of the 10,000 shRNA pool, and the set of validated positive and negative control shRNAs described above were added to each pool. When the 2,000 shRNAs represented in all three pools were examined, there was a high correlation between different pool sizes (Figure 7a). Furthermore, five out of six positive control hairpins were identified as hits in all three screening pool sizes (Figure 7b). There was a clear distinction between validated positive and negative control scores in all screens (Figure 7b). However, there was a larger than expected variation in the negative control score, with validated controls changing representation by up to 25%. This effect was also seen in the distribution of 101 non-targeting shRNAs within the 10,000 shRNA pool (Figure 7c). Furthermore, the depletion observed in positive control hairpins was smaller than expected based on single hairpin GFP competition assays (Figure 7b).

These results suggested that the scoring system performs consistently regardless of pool size and the screen is as sensitive in a 10,000 shRNA pool as in a 2,000 shRNA pool. Therefore, to maximize throughput and minimize expense, we decided to use 10,000 shRNA pools for screening. Since the genome-wide library encompasses approximately 60,000 reagents, we reasoned that a pool size of approximately 10,000 shRNAs would be an ideal choice for this library. This would enable a genome-wide screen to be run on a single eight-lane Illumina flow-cell along with appropriate sequencing controls. Furthermore, a 10,000 shRNA pool size would require approximately 15,000,000 cells to be maintained in tissue culture over the course of the experiment, assuming an MOI of 0.7 and a representation of 1,000 cells per hairpin. This pool size enabled us to maintain the simplicity and reproducibility of the associated tissue culture work, which might become cumbersome in larger pool sizes.

To establish the reproducibility of the screening methodology, we repeated the screen for cell viability in MCF7 cells in a total of four biological replicates. The replicates showed high correlation despite being performed by different researchers at different times, suggesting that the screening method is robust (Figure 7d). Furthermore, >98% of library hairpins were identified in all screen replicates, compared to 49% of half-hairpin probes and 82% of barcode tiling probes having intensity above background in microarray-based approaches [10]. This suggests a greatly improved sensitivity for the NGS profiling approach.

## Conclusions

Here we describe detailed and optimized methods for high-throughput shRNA screening using NGS. Using massively parallel sequencing in place of microarray hybridization for deconvolution of shRNA barcode screen results offers several advantages. Firstly, we demonstrate that NGS profiling offers greater sensitivity, enabling more hairpins to be detected above background. Secondly, we show that NGS profiling has a better dynamic range when compared to literature examples of microarray-based approaches, since sequencing cannot be easily saturated in the same way that hybridization can, thus enabling a greater separation of true effects from background noise. Thirdly, sequencing of tags is both scalable and flexible, enabling new hairpins to be incorporated into the work schema without having to print a new batch of custom microarrays. Finally, our computational pipeline offers the ability to identify sequences where bases are not called due to sequencing errors, or mutated during PCR, since short reads can be matched inexactly to the reference library of shRNA barcode sequences.

We do note that one of the major limitations of any shRNA library is the efficacy of gene silencing, an issue we have not addressed here. However, the ability to rapidly assess silencing capacity and thus develop algorithms that would predict effective gene knockdown is improving [12]. In addition, an increase in the redundancy of shRNA libraries (the number of different shRNA constructs per gene) will also improve the general effectiveness of pooled RNAi screens. Finally, improvements in sequencing technology will undoubtedly increase read number per lane; these developments will thus enable the use of larger shRNA pool sizes that could accommodate libraries with higher levels of redundancy. Nevertheless, even with the existing commercial shRNA libraries and also the ever-increasing availability of cost-effective NGS, the methods we describe here should enable the wider applicability of this powerful technology.

## Materials and methods

### shRNA library

Although the following methods are suitable for most viral shRNA libraries, the work described here used the Thermo Scientific Open Biosystems GIPZ Lentiviral human shRNAmir library (version 2). These methods could be used for other shRNA libraries, such as the RNAi consortium library [13], with altered PCR and sequencing primers. The GIPZ Lentiviral human shRNAmir library we used encompasses 61,416 distinct hairpin constructs targeting 15,739 human protein coding genes (based upon the Ensembl 56 build). In the GIPZ vector the 19-nucleotide siRNA sense sequence is inserted into a human mir-30 backbone [14]. shRNA sequences are designed to have destabilized 5' ends in the antisense strand to encourage stand-specific incorporation into the RISC [14]. The vector backbone includes a GFP-coding sequence that is transcribed as part of a bicistronic transcript with the shRNA sequence allowing the visualization of shRNAmir expressing cells, and a puromycin resistance marker for selecting infected cells.

### Bacterial culture

LB media (1 ml) containing 50 μg/ml ampicillin was added to 96-deep-well microplates using a multidrop (Thermo Fisher, Waltham, MA, USA), and 1 μl of each bacterial inoculant (extracted from fully thawed 96-well glycerol stocks) was seeded per well using a Beckman FX robot. Culture plates were stacked evenly in shaking incubators (200 rpm) ensuring even air circulation. Following 16 hours of incubation at 37°C, cultures from a single batch of plates were pooled and plasmid DNA was isolated using a Plasmid Maxi kit (Qiagen, Crawley, UK) and normalized to a standard concentration (0.5 μg/ml). Plasmid DNA pools were combined in equal concentrations to create screening pools of different complexities.

### Lentiviral packaging

For both calcium phosphate- and lipid-based transfection using lipofectamine 2000, 293T cells were seeded onto 10 cm dishes (50 to 80% confluent), and co-transfected with the desired shRNA library pool, along with the packaging plasmids psPAX2 and pMD2.G [15], in a Safety Category II facility. Thirty-six hours post-transfection, supernatant was collected, supplemented with 4 μg/ml polybrene and filtered through a 0.45 μm membrane. Viral supernatant from multiple 10 cm dishes was pooled, aliquoted and stored at -80°C for future use. Determination of viral titer allowed subsequent infection of screening cell lines at intended efficiencies. For this, both target and reference 293T cells were infected with 1:2 serial dilutions of the virus. Virus was removed 24 hours later and cells incubated for a further 48 hours at 37°C, after which the proportion of GFP-positive cells was determined by fluorescence-activated cell sorting (FACS) to provide an estimate of the fraction infected and viral titer.

### Viral transduction and cell sampling

Cells were infected with viral pools using a MOI of 0.7 and media replenished after 16 hours. Seventy-two hours post-infection, when viral integration was presumed complete, cells were exposed to 1 mg/l puromycin for 2 days to select for cells with viral integration. Following puromycin selection, cultures were divided into two or more replica cultures, and continuously cultured for 2 to 3 weeks, dividing cultures regularly to ensure continued logarithmic growth and to maintain hairpin representation at approximately 1,000 cells per shRNA [4,5]. Cells were harvested in suspension and either stored frozen at -20°C or processed immediately as described below.

### Barcode recovery

Genomic DNA extraction and purification from cultured cells was carried out using a Gentra Puregene kit (Qiagen). shRNA sequences integrated into genomic DNA were recovered by PCR amplification using the following primers and a procedure based upon that described by Hannon, Elledge and Lowe [4,5]: p5+mir30, 5'-AATGATACGGCGACCACCGACTAAAGTAGCCCCTTGAATTC-3'; p7+Loop, 5'-CAAGCAGAAGACGGCATACGATAGTGAAGCCACAGATGTA-3'. PCR was performed on PTC-225 3DNA Engine Tetrads (Bio-Rad, Hemel Hempstead, UK) using Amplitaq Gold polymerase and 20 to 33 cycles of denaturation (95°C, 30 s), annealing (52°C, 45 s) and extension (72°C, 60 s). In general, 2 μg genomic DNA was used per 100 μl PCR reaction and 48 PCR reactions were performed per 10,000 shRNA pool. PCR products from multiple parallel reactions were subsequently pooled, concentrated and purified using a QIAquick gel extraction kit (Qiagen).

### Quantitative PCR DNA quantification

For absolute DNA quantification, real-time PCR was performed using two alternative strategies: SybrGreen and TaqMan. Both approaches used the appropriate universal 2X PCR mix from Applied Biosystems and the following oligonucleotides at 250 nM each: P5, AATGATACGGCGACCACCGA (20-mer); and P7, CAAGCAGAAGACGGCATACGA (21-mer). For TaqMan only, 250 nM of a 21-mer DLP was included (CCCTTGAATTCCGAGGCAGTA), with 5' reporter (6FAM fluorescein) and 3' quencher (tetramethylrhodamine (TAMRA)) as detectors, and ROX passive reference dye. Forty-cycle measurements of triplicate 25 μl reactions containing 10% template sample at a theoretical concentration of 10 pM (as defined by Agilent Bioanalyzer) were carried out in an ABI 7900 instrument. Solexa library concentrations were then inferred by comparing measurements to the standard curve using the Sequence Detection System (SDS) v2.2.1 software. To generate the standard curve, a 10-fold dilution series of a standard 100 nM sample (7.7 ng/μl for 117 bp) was used (final range 0.1 to 100 pM).

### Barcode sequencing

Following quantification, denatured shRNA-seq libraries (3 pM in NaOH) were pumped through eight-lane flowcell channels using the cluster station of an Illumina GAIIx sequencing platform. Bridge PCR was executed using the manufacturer's protocol (Amplification-linearization-blocking and multiple primer hybridization version 3). The sequencing primer (TAGCCCCTTGAATTCCGAGGCAGTAGGCA) was designed to sequence from two bases upstream of the 19 bp shRNA sense sequence. Twenty-six cycles of sequencing-by-synthesis (single read) were performed on an Illumina GAIIx according to the manufacturer's protocol (version 4).

### Image analysis and base calling

Raw image data were analyzed using GA pipeline v1.4. PhiX was run as a control to ensure correct phasing. Base calling was performed by the Bustard package using the Chastity filter with a threshold of 0.6. The Chastity filter was applied to bases 3 to 21 of the 26 bases sequenced (the shRNA sense sequence) only. A maximum of two uncalled bases were allowed in these 19 bases. FASTQ files from this study are available at the ROCK web page [16] and at the European Nucleotide Archive [17].

### shRNA library alignment

FASTq files generated by the GA pipeline were mapped to reference shRNA libraries using a bespoke Perl program, shALIGN. shALIGN trims short reads to the 19 bp sense sequence based on user-defined base positions, and groups identical reads disregarding sequence quality scores. shALIGN employs a hamming distance algorithm to align binned short read sequences to a user-defined reference shRNA library provided in a standard tab-delimited text format. The user is able to specify the maximum number of mismatches permissible between the short read and the reference. This alignment is equivalent to using Bowtie with the flowing flags: --best --strata -v 2 -m 1 -a. However, using shALIGN negates the need to construct and index a specific Bowtie reference library by inserting the sense sequence of each shRNA into a longer sequence. It also saves the need to write a script to parse the Bowtie output to count the number of reads mapped to each library construct at each edit distance. For the GIPZ library this was set at two mismatches as the vast majority of library sequences were separated by an edit distance of greater than two. All short reads that match to more than one library sequence at the same distance were excluded from further analysis, and logged as ambiguous. The shALIGN program outputs the total number of short reads matching to each library hairpin in each lane. We routinely load screen results into the ROCK breast cancer functional genomics database [18], where screen reagents are fully annotated to the latest genome build. ROCK provides a mechanism for the sharing and publication of raw and processed RNAi screen results. Source code and linux binaries for the shALIGN program are available from the ROCK web page [16].

### Statistical analysis

To facilitate statistical analysis of screen results, we have developed a novel R package, shRNAseq. This package is based on the NChannelSet class in Bioconductor [19], originally designed to handle microarray gene expression data, and serves as a single user-friendly package encompassing all of the steps required. shRNAseq reads in matrices of short read counts per shRNA construct, generated by shALIGN, along with annotations of shRNA constructs and sequenced samples.

The package is designed to compare a pair of related screen conditions. For example, in a viability screen one would compare the hairpin representation in the starting population (which could be from the plasmid, virus or an early time point post-viral integration) to the hairpin representation at the end of the screen (for example, after 2 weeks). Alternatively, for a drug screen one would compare the drug and vehicle treated arms at the same time point. The package can analyze multiple screen replicates simultaneously. Each screen condition is loaded into the package separately, and annotated using files describing the shRNA constructs and the sample treatments. After data loading, read counts per hairpin are log2 transformed and then the ratio of the two screen conditions is calculated. This log ratio is normalized using a loess fit to the log mean read count for each screening pool. The distribution of normalized scores per pool is then rescaled by dividing by the pool MAD. If appropriate, screen biological replicates can be quantile (rank) normalized to ensure identical distributions, and scores can be summarized across replicates using a variety of methods (for example, median, regularized *t*-test). Finally, the package plots screen distributions before and after normalization and reports a table of normalized scores. The R package is accompanied by a detailed vignette describing the methodology and usage at the ROCK web page [16].

Hit detection was performed using three different methods. In the first method, replicate scores for each hairpin were summarized using the median and a hit threshold estimated from a quantile-quantile plot, identifying hairpin scores that significantly differed from the normal distribution. Second, the Gene Set Analysis R package [20] was used to look for enrichment or depletion of sets of hairpins targeting the same gene. This approach makes use of the hairpin redundancy within the library and works best in libraries containing multiple hairpins per gene. Finally, the RIGER algorithm [21] in the GENE-E java package [22] was also used to look for enrichment of shRNAs targeting the same gene. Here we used the log fold change metric and weighted average method. Source code and documentation for the shRNAseq R-package are available from the ROCK web page [16].

### Supplementary methods

Full methods, including a detailed screening manual, are provided in Additional file 4.

## Abbreviations

DLP: dual-labeled probe; GAIIx: Genome Analyzer IIx; GFP: green fluorescent protein; MAD: median absolute deviation; miRNA: microRNA; MOI: multiplicity of infection; NGS: next generation sequencing; qPCR: quantitative PCR; RISC: RNA-induced silencing complex; RNAi: RNA interference; RNASeq: RNA sequencing; shRNA: short hairpin RNA; shRNAmir: shRNA encoded within a miRNA; siRNA: short interfering RNA; TU: transducing unit.

## Competing interests

The authors declare that they have no competing interests.

## Authors' contributions

DS performed screens, analyzed the data, designed the software, implemented the R package and drafted the manuscript. CJL, AMP, JF, DB, CL, NM, CMI and MAC developed laboratory methods and/or performed screens. CM, JH and MZ implemented the shALIGN software. KF, IA and IK performed the sequencing. CJL and AA conceived the study and experiments and drafted the manuscript. All authors read and approved the final manuscript.

## Supplementary Material

Additional file 1 **Engineered depletion of shRNAs**. To establish the sensitivity of the screening system, we performed a series of engineered depletion experiments. We manually altered the representation of constructs in a 10,000 shRNA screening pool so that approximately 1,000 hairpins were depleted by 75%, approximately 1,000 depleted by 50% and approximately 1,000 depleted by 25%. Click here for file

Additional file 2 **Detection of hairpin depletion at reduced read counts**. Reads were sampled at random from an engineered depletion experiment involving approximately 10 million reads to give datasets of either approximately 5 million or approximately 2.5 million reads in total. shRNA depletion was estimated from these new datasets to show that depletion of 50% could be observed in datasets containing approximately 2.5 million reads. Click here for file

Additional file 3 **Positive and negative controls for the MCF7 viability screen were established using a single hairpin GFP-competition assay**. The bar chart indicates the proportion of GFP positive cells remaining after 2 weeks of culture. The bar represents the average from three biological replicates. The error bars indicate the standard deviation. Click here for file

Additional file 4 **Detailed shRNA screening protocols**. This Word document describes in detail all of the steps of the shRNA screening protocol from library generation to massively parallel sequencing. Click here for file
