# Supplementary material for: High-throughput RNA interference screening using pooled shRNA libraries and next generation sequencing
Source: Genome Biol. 2011 Oct 21;12(10):R104. doi: 10.1186/gb-2011-12-10-r104 (PMC3333774; doi:10.1186/gb-2011-12-10-r104)
Supplement: Additional file 4 — Detailed shRNA screening protocols. This Word document describes in detail all of the steps of the shRNA screening protocol from library generation to massively parallel sequencing. [file gb-2011-12-10-r104-S4.DOC]

**Table of Contents**

Standard Operating Procedure (SOP) for shRNA screening

1. Lentiviral PRODUCTION [3](#__RefHeading___Toc107655704)

1.1 Materials [3](#__RefHeading___Toc107655705)

1.2 Procedures: CaPO_4_-mediated DNA transfection & virus collection [6](#__RefHeading___Toc107655707)

2. Lentiviral Stock TITRATION [8](#__RefHeading___Toc107655708)

3. shRNA Screen Generic Procedure: Transduction & Sampling [10](#__RefHeading___Toc107655709)

4. Genomic DNA Preparation & Solexa Sample Preparation for shRNA representation studies [12](#__RefHeading___Toc107655710)

4.1 Genomic DNA isolation [12](#__RefHeading___Toc107655711)

4.1.1 Preliminary Considerations [12](#__RefHeading___Toc107655712)

4.1.2 Procedure [12](#__RefHeading___Toc107655713)

4.2 PCR AND SOLEXA SAMPLE PREPARATION [15](#__RefHeading___Toc107655714)

4.3 Solexa PCR Product Quantitation [19](#__RefHeading___Toc107655715)

4.3.1 Bioanalyzer shRNA Sample Quantitation [19](#__RefHeading___Toc107655716)

4.3.2 qPCR for Solexa Library Quantification [20](#__RefHeading___Toc107655717)

5. Solexa Run [26](#__RefHeading___Toc107655718)

# 1. Lentiviral PRODUCTION

## 1.1 Materials

Per transfection:

**10ug GIPZ shRNA plasmid (or plasmid pool)**

**4.4ug psPAX2 packaging plasmid**

**1.8ug pMD2.G packaging plasmid**

(recommend making stock mixture of 440ug psPAX2 and 180ug pMD2.G for 100 transfections)

For each new batch of packaging plasmids, check, using a small number of plates, that sufficiently high titre virus is produced. In case of failure, plasmid quality can be checked by restriction digest.

**Quality Control Restriction Digest Checks** to ensure integrity of unrecombined plasmids or cloning success.

**- pGIPz backbone:** SacII digest renders 3 fragments of the following sizes 1259, 2502, 7927 bp

**- pGIPz confirmation of shRNA harbouring:**

EcoRI + XhoI digest should release a 117 bp insert.

**- psPAX2 & pMD2.G backbones**: EcoRI double cutter for both, renders (i)1.7 & 4.3 Kb, and (ii)4.4 & 6.6 Kb fragments respectively.

**For more information, see:**

GIPZ plasmid:

http://www.openbiosystems.com/collateral/rnai/pi/pGIPZ_manual.pdf

psPAX2 Plasmid:

<http://www.addgene.org/pgvec1?f=c&cmd=findpl&identifier=12260>

pMD2.G Plasmid

<http://www.addgene.org/pgvec1?f=c&identifier=12259&atqx=pmd2.g&cmd=findpl>

## 1.2 Procedures: CaPO_4_-mediated DNA transfection & virus collection

Summary: This protocol is optimised for cells in 10cm plates. Any method is suitable for transfecting 293T cells, e.g. Lipofectamine 2000.

Below is a protocol for CaPO_4_-mediated transfection.

Thaw 293T cells and grow them in DMEM + 10%FBS + Gln + PenStrep until the required number of cells is reached (2-5 passages depending on how much virus is required). The day before transfection count the cells once with the cell counter and plate them at 1.5x10^6^/10cm plate (the number of plates depends on how much virus is necessary). 293T should be 50-80% confluent at transfection (approx 24 hours after plating).

1. For each transfection, make up a ‘buffer’ solution and a ‘DNA’ solution in FACS tubes.

Buffer solution:

500ul 2X HBS

DNA solution:

10ug pGIPZ shRNA plasmid DNA

psPAX2/pMD2.G packaging plasmid mix

62.5ul 2M CaCl_2_

Sterile H_2_O up to 500ul total volume.

1. Bubble air through the buffer solution using a 1ml disposable pipette + pipetteaid. Add DNA solution drop-wise to the bubbling buffer solution using a bulbed Pasteur pipette until the DNA solution is finished.
2. Leave for 10 minutes at room temperature. Solution should turn slightly cloudy (CaPO_4_/DNA ppt). Large, visible clumps indicate insufficient mixing.
3. Aspirate old medium off packaging cells. Replace with 9ml fresh, pre-warmed medium.
4. Retain 1ml pipette from above. Add whole 1ml transfection solution on top of culture medium. View on inverted microscope: grainy flakes should float down onto the plate surface. Incubate as normal for 16 hours.
5. At 16h post-transfection, change medium to 6ml collection medium (medium suitable for target cells to be infected: usually DMEM-based medium, as above)
6. Lentiviral supernatant is collected and filtered starting 36 hours post-transfection, every 24 hours for 3 total collections, replenishing 293T plate with 6ml fresh medium after each collection. For each collection add 6ul polybrene (4mg/ml stock), suck off supernatant into 10ml syringe. Filter through a 0.45um filter into a 15/50ml falcon. Repeat for each virus collection.
7. Supernatant can be used immediately on target cells or stored in 15ml/50ml falcon tubes or 2ml cryovials at 4ºC (few days) or -80ºC (indefinitely). Preferred procedure for screening is to store all filtered supernatant at 4ºC until the final collection, then pool all virus (for reproducibility). Store pooled virus in 15ml Falcon tube aliquots at -80ºC. Thaw an aliquot for titre determination (below).
8. Discard transfected 293T plates once all required virus collected.

**Caution:** Disinfect 293T plates (and any other virus-contaminated plasticware) in 5% Trigene overnight before discarding.

1. Titre virus on target cell lines, if required, in order to achieve **optimal infection efficiency**: typically 70% GFP^+^ for shRNA screens. (Balance between maximising infection efficiency for maintaining hairpin representation and enhancing genomic PCR efficiency, while limiting multiple integration events per target cell.

# 2. Lentiviral Stock Titration

Summary: Determination of viral titre allows subsequent lentiviral infection at intended infection efficiencies, using informed dilutions of lentivirus.

1. The day before transduction, count 293T with the cell counter and seed 24 well plates with target cell line (e.g. MCF7 cells) at 5x10^4^ cells per well. Plate extra wells so that at the time of transduction, the cells from one well can be counted for a more accurate cell number.
2. After 24h, count the cells in one 24-well.
3. Prepare the 1 in 3 serial dilutions of the virus in media.
4. Remove the media from the 24 well plates and add the 400ul virus dilutions to each required well starting with 400ul of neat virus. Repeat for 5 dilutions.


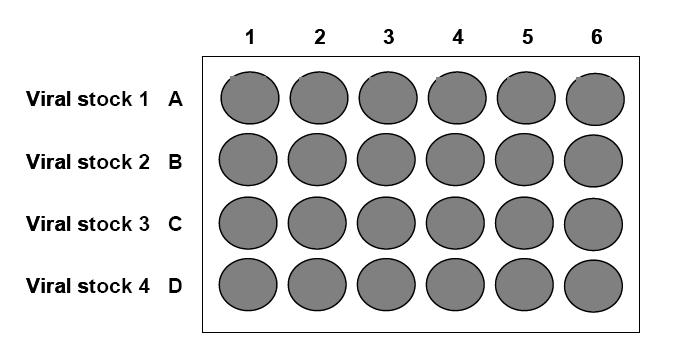


1. Incubate for 24h at 37°C, 5% CO_2_. Replace the virus with 1ml fresh media.
2. Incubate for a further 48h at 37°C, 5% CO_2_. Measure the %GFP by FACS for each of the wells counting 5000 events.
3. Calculate the titre expressed as transducing units (TU)/ml

Transducing units = (F x C/V) x D

F= frequencing of GFP positive cells (% divided by 100)

C= total number of cells in well at time of transduction

V= volume of inoculum in ml (400ul = 0.4ml)

D= lentivirus dilution

The applied Biosystem protocol suggests using for the virus titre a % GFP that falls within the range of 1-30% (where the standard curve is linear) to calculate the most accurate titre, in order to use a concentration that is not saturating. Typical titres range from 2 x 10^5^ to 2 x 10^7^ TU/ml.

(The 1-30% GFP covers the linear part of the standard curve for the dilutions and therefore using the slope to calculate the virus titre will give a similar value).

To obtain optimal expression of your hairpin, the lentiviral stock will need to be transduced using a suitable MOI. MOI is defined as the number of viral particles per cell and generally correlates with the number of integration events and as a result expression. Typically an MOI of ~1 is used but optimal MOI can vary depending on the target cell line.

# 3. shRNA Screen Generic Procedure: Transduction & Sampling

Summary: The screening procedure post-infection, pre-genomic DNA preparation/PCR is the most variable and dependent on the type of screen regarding e.g. cell lines, timescales, presence/absence of drugs and their doses. Below is a sample protocol for a generic ‘straight lethal’ screen for identifying lethal shRNAs in a drug-free setting.

Throughout the screen the representation needs to be kept at 1000cells/hairpin. For a 1k pool the number of infected cells needs to be kept above 1x 10^6^ cells (plate at least 2x10cm) and 1x 10^7^ cells for a 10k pool (plate at least 2xT175cm flask).

1. Thaw target cells and passage them for 2-4 times until the required number of cells to start the screen is reached. For best reproducibility, thaw the same passage of cells for each replica screen and use them at the same passage after thawing for the different replicas.
2. Day 0. Plate enough cells to give a final representation of 1000 cells/hairpin and allow to adhere O/N. (For 1k pool plate at least 2 x 10^6^ cells assuming infection efficiency is above 50%, for 10k pools, plate at least 2x10^7^ cells.)
3. Day 1. Remove the medium and add the virus to an MOI of 1 containing 4 ug/mL Polybrene. Take a sample of viral pool and extract RNA to check for hairpins present at this stage.
4. Day 2. 16h post infection change the media.
5. Day 3. Trypsinise each of the infected plates (the amount of trypsin and medium added varies upon the size of the plate). Mix together the cell suspension and plate into double the amount of original dishes. This is done because we need to take half of the amount of the infected cells to make genomic DNA for the T0 (72h post infection).
6. Day 4. Trypsinise half the dishes. Keep 0.5ml for FACS analysis, count the cell number as before to make sure representation is kept at 1000cells/hairpin, spin down at 1500rpm for 5’ and freeze the pellet for T0 at -20. Replate the other half of the cells in culture, add drug if required.
7. Every 3 days replace with freshly prepared media (with or without drug if required) and passage the cells when 70% confluent making sure that the number of infected cells is kept as required for 1000 cells/hairpin. The frequency of passaging depends on how quickly the cells divide.
8. 5 population doublings after the T0, trypsinise the cells, mix together the cell suspension (keeping each drug treatment separate), take 0.5ml for FACS, remove half the volume to pellet and freeze, then plate the remaining cells, as done for T0. Count the cells to make sure that representation is kept both in the cell pellet and the cells remaining in culture.

N.B. when passaging the cells in the days leading up to each time point, make sure there are at least double the amount of plates required for representation so half can be removed and half can be passaged on.

1. Repeat step 8 at each required time point.
2. Extract genomic DNA from frozen cell pellets. (see following protocol)

**Use same volumes of media and/or other reagents and cultures conditions for all the screen replicas to ensure reproducibility.**

# 4. Genomic DNA Preparation & Solexa Sample Preparation for shRNA representation studies

Adapted from Silva *et al*., Science 2008.

## 4.1 Genomic DNA isolation

### 4.1.1 Preliminary Considerations

Perform all the steps in a plasmid DNA-free environment. Avoid any possibility of miR30 contamination. Ideally perform all the steps in a template-free PCR lab! Don’t share any solutions, tips or equipment with standard plasmid work.

Use Gentra Puregene kit for genomic DNA isolation from cultured cells.

Use reagent volumes depending on number of cells, e.g. Choose ■ if processing 1–2 x 10^6^ cells; choose ▲ if processing 1–2 x 10^7^ cells, or use multiples of these volumes if processing different cell numbers.

**Protocol: DNA Purification from Cultured Cells Using the Gentra Puregene Cell Kit**

Summary: This protocol is for purification of genomic DNA from 1–2 x 10^6^ or 1–2 x 10^7^ cultured cells using the Gentra Puregene Cell Kit.

**Below is the protocol starting from a frozen cell pellet.**

Significant Changes to the handbook protocol appear in red.

### 4.1.2 Procedure

1. Harvest cell pellet as usual: cell trypsinisation, resuspension in media (volumes will depend on cell number), take aliquot for cell count. Remainder: spin 1500 rpm for 5 minutes, aspirate medium, freeze pellets at -20ºC.

*****RECOMMENDED: SAVE ALL FROZEN PELLETS FROM shRNA SCREEN FOR SIMULTANEOUS gDNA EXTRACTION AT THE END OF THE SCREEN*****

2. Thaw cell pellets on ice

3. Vortex the tube vigorously to resuspend the cells in the residual supernatant**.** Vortexing greatly facilitates cell lysis in the next step.

4. Add ■ 300 μl or ▲ 3 ml Cell Lysis Solution to the resuspended cells and pipette up and down or vortex on high speed for 10 s to lyse the cells.

INCUBATE OVERNIGHT at 37ºC TO ENSURE COMPLETE CELL LYSIS.

During the day agitate periodically [e.g. hourly on Thermomixer (■) or manually during waterbath incubation (▲)] and then leave it overnight.

Incomplete cell lysis will reduce DNA yield. Lysed sample should no longer have visible cell debris or excessive viscosity.

Samples are stable in Cell Lysis Solution for at least 2 years at room temperature (15–25°C).

5. Required: For RNA-free DNA, add ■ 1.5 μl or ▲ 15 μl RNase A Solution, and mix by inverting 25 times. Incubate for 5 min at 37°C. Incubate for ■ 1 min or ▲ 3 min on ice to quickly cool the sample**.** Sample can be incubated at 37°C for up to 1 h.

6. Add ■ 100 μl or ▲ 1 ml Protein Precipitation Solution, and vortex vigorously for 20 s at high speed.

7. Centrifuge ■ for 1 min at 13,000–16,000 x g or ▲ 10 min at 2000 x g

(For 15ml Falcon Tubes (▲) recommend 4500rpm for 10 min in swing-bucket centrifuge) The precipitated proteins should form a tight pellet. If the protein pellet is not tight, incubate on ice for 5 min and repeat the centrifugation.

8. Pipet ■ 300 μl isopropanol into a clean 1.5 ml microcentrifuge tube or ▲ 3 ml isopropanol into a clean 15 ml centrifuge tube and add the supernatant from the previous step**.** Be sure the protein pellet is not dislodged during pouring.

9. Mix by inverting gently 50 times.

10. Centrifuge for ■ 1 min at 13,000–16,000 x g or ▲ 3 min at 2000 x g.

(For 15ml Falcon Tubes (▲) recommend 4500rpm for 10 min in swing-bucket centrifuge) The DNA will be visible as a small white pellet.

11. Carefully discard the supernatant, (Use of 1ml Gilson pipette rather than vacuum aspirator is recommended to avoid sucking up pellet). Drain the tube by inverting on a clean piece of absorbent paper, taking care that the pellet remains in the tube.

12. Add ■ 300 μl or ▲ 3 ml of 70% ethanol and invert several times to wash the DNA pellet.

13. Centrifuge for ■ 1 min at 13,000–16,000 x g or ▲ 1 min at 2000 x g.

(For 15ml Falcon Tubes (▲) recommend 4500rpm for 5 min in swing-bucket centrifuge)

14. Carefully discard the supernatant. Use of 1ml Gilson pipette rather than vacuum aspirator is recommended to avoid sucking up pellet. Drain the tube on a clean piece of absorbent paper for 5 s, taking care that the pellet remains in the tube. Wipe the residual ethanol with a tissue Allow to air dry. The pellet might be loose and easily dislodged.

Pellet should be dry (free from 70% ethanol), but not overdry (inhibits DNA resuspension). Recommend leaving tube 5 minutes, then use a Gilson pipette to suck droplets of 70% ethanol from the sides of the tube.

15. Add ■ 100 μl or ▲ 500 μl DNA Hydration Solution (volumes doubled to aid hydration) and vortex for 5 s at medium speed to mix. Transfer partially resuspended DNA to a 1.5ml microfuge tube if currently in 15ml Falcon tube (▲).

16. Incubate at 65°C for 1 h to dissolve the DNA, with 1000rpm agitation on Thermomixer.

17. Incubate overnight at 37°C on Thermomixer with intermittent mixing (e.g. 1500rpm for 30s every hour). Ensure tube cap is tightly closed to avoid leakage.

18. Centrifuge briefly (5 seconds full speed) to spin down solution on underside of tube lid. No white DNA pellet should be seen if resuspension successful.

19. Nanodrop 2ul sample to check for yield and purity (typically 0.5 – 2 ug/ul).

(Optional: To view DNA degradation / relative yield, run 5ul samples on 0.5% agarose TAE gel.)

20. Store samples at 4°C short term or -20°C long term. Avoid repeated freeze-thaw cycles.

Fixed Cells

## 4.2 PCR and Solexa Sample Preparation

Summary: This PCR procedure amplifies the half-hairpin sequences integrated into the genomic DNA. Primers contain the P5 and P7 adaptor sequences at their 5’ ends, hence adaptor incorporation occurs during PCR, eliminating the need for subsequent adaptor ligation.

To keep representation for each sample/template you have to perform multiple PCR reactions in parallel (depending on the complexity of the pool studied).

A diploid human cell contains ~6 pg genomic DNA

1 ng genomic DNA contains the DNA from 167 diploid cells

*e.g. to keep 1000fold representation of a pool of 1000 shRNAs at MOI=1 you need to use genomic DNA from 1,000,000 cells (i.e. 6* μ*g) as PCR template. In each PCR you can use a maximum of 2* μ*g.*

**Primers:**

p5+**mir3**: AATGATACGGCGACCACCGA**CTAAAGTAGCCCCTTGAATTC**

p7+**Loop**: CAAGCAGAAGACGGCATACGA**TAGTGAAGCCACAGATGTA**

**AIM FOR 2ug gDNA PER 100ul REACTION**

(adjust input gDNA concentration or volume to achieve this.)

**Reaction:**

Template gDNA (~1 μg/μl) 2ul

10xPCR-buffer (MgCl2 not included) 10ul

25mM MgCl2 6ul

Primer p5+mir3 (10μM) 5ul

Primer p7+loop (10μM) 5ul

dNTP (10 mM each) 2ul

DMSO 2ul

Amplitaq Gold 0.5ul

H_2_O HPLC 67.5ul

1. Prepare a mastermix (without template) for (reactions+2) samples.
2. Aliquot 98ul mastermix (without template) to each PCR tube. Add 2ul template. Always run a parallel negative control (98ul mastermix without template)

**Cycling:**

95°C 5 min

95°C 30s )

52°C 45s ) 33 cycles

72°C 60s )

72°C 10 min

4°C ∞

**Test gel**

Run a 10ul aliquot on a 2.5% agarose TAE gel The correct PCR fragments have a length of 117bp. No band should be seen in the negative control.

**Product Concentration:**

Use the Qiagen gel extraction kit (without running gel) to concentrate and cleanup fragments. The gel extraction kit is more effective than PCR cleanup or Nucleotide removal kits for high yield of small PCR products.

1. Combine all parallel PCRs up to maximum of 2ml total volume.
2. Add 3 volumes buffer QG, then 1 volume isopropanol
3. Vortex
4. Pass through purple gel extraction column (will take >1 spin if large volume) (Use of vacuum pump facilitates this step).
5. 0.75ml PE through column
6. Empty collection tube and spin additional 2.5 min at full speed.
7. Transfer column to clean 1.5ml tube.
8. Elute in 40ul EB.

**Gel purification of concentrated PCR products.**

Run concentrated PCR samples on a 2.5% agarose TAE gel with 4ul of Ethidium bormide (10mg/ml stock) in 200ml gel. Run at 130V for 1h. Always keep one well empty between sample lanes to prevent cross contamination.

Analyze gel under a UV lamp and cut out the correct bands (117 bp is correct length). Use clean tips to load the gels, and clean blades to cut the bands.

**Gel extraction with QIAquick gel extraction kit (Qiagen).**

Follow manufacturer’s protocol, except for the following modifications:

Do not heat gel slab at any point. Instead solubilise in QG at room temperature with agitation on plate shaker.

1. Wash column a second time with 0.75 ml PE.
2. Elute DNA in 35 μl EB.
3. Determine the DNA concentration with preferred method [e.g. DNA 1000 Bioanalyser Chip, Custom Solexa qPCR – see following protocols].

**Examples of gel images:**

Solexa PCR products: Test Gel (Pre-concentration)

[no product in no template control (right lane)]


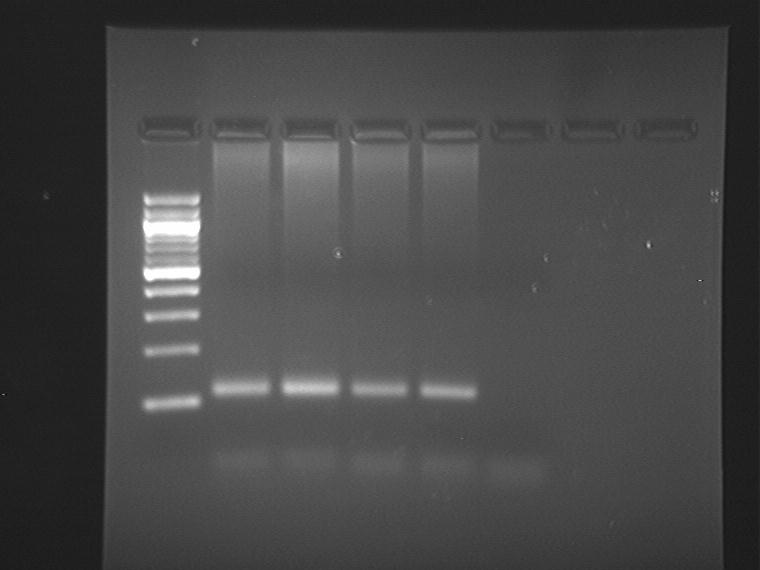


Solexa PCR products: Post-concentration


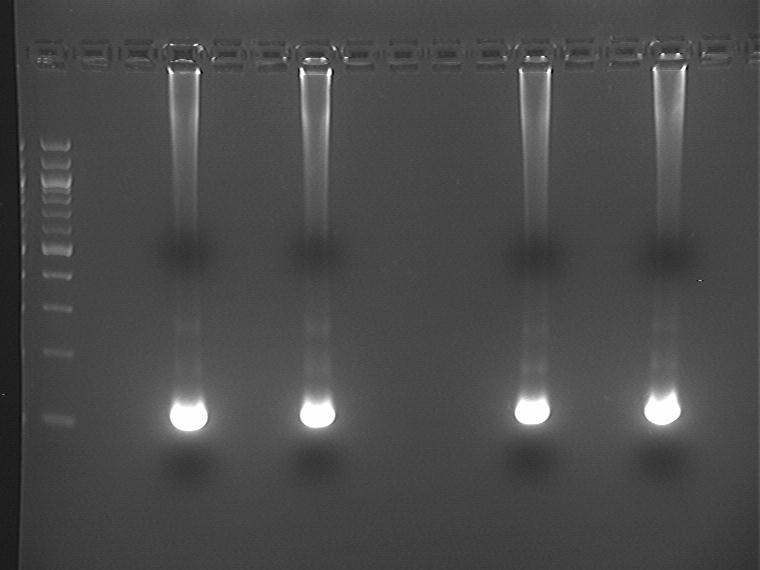


**Specificity Demonstration**

Products on left half of gel are expected products. Note that these primers are specific for the retrovirally-transduced MiR-30 constructs and not endogenous MiR-30 (no product from untransduced cells). Right half of gel is for illustration purposes only: use of non-discriminating generic MiR-30 primers would amplify both endogenous MiR30 and transduced MiR30.

##

## 4.3 Solexa PCR Product Quantification

Summary: Accurate quantification of Solexa samples is required for achieving optimal cluster densities during cluster generation. (Cluster density should be optimised to give maximal number of sequence reads, without excessive, deleterious cluster overlap

### 4.3.1 Bioanalyzer shRNA Sample Quantification

After running the samples on the DNA 1000 chip:

There will normally be two peaks present in a shRNA sample preparation at 125-130bp and 185-190bp.

Due to the size of the one or both of these peaks, they may not be recognised by the software. If this is the case manual integration must be carried out.

Viewing Results

To view results click ‘Data’ icon on left of bioanalyzer window.

Click ‘electropherogram’ tab and click a sample to view. If one or both peaks have not been recognised proceed with manual integration. If all the peaks have been recognised nothing more needs to be done.

Manual Integration

While viewing a single result ‘right-click’ mouse while on the electropherogram and select ‘Manual Integration’

Draw a box to Zoom to the area where the peak hasn’t been recognised, then ‘right-click’ mouse to select ‘Add Peak’

Adjust the new peak by clicking and dragging the solid circles at either end of the blue line of the new peak. Taking care to make sure this blue line remains flat on the baseline.

The circle will automatically ‘stick’ to the lines of the electropherogram. To adjust the circles away from the lines press and hold the ‘Ctrl’ key while moving the circles.

**ATTENTION**: It is crucial to have the blue line of the peaks flat on the baseline to gain accurate quantitation.

Final Quantitation Results

Once Manual integration has been performed (if necessary) add the concentration of both peaks together to get the final concentration of the sample.

### 4.3.2 qPCR for Solexa Library Quantification

(via Absolute Quantification. Relative ΔCt also plausible)

# **MATERIALS**

**- Universal 2X PCR mix** Part No. 4309155 (Sybr-Green) & Part No. 4304437 (TaqMan) from Applied Biosystems.

These could be put together in-house by combining with the following ingredients: AmpliTaq Gold (0.1 U/uL), 2x PCR buffer (1:5 10x), 5 mM MgCl_2_, 500 uM each dNTP, 30 uM ROX passive reference dye for internal normalisation, 6% DMSO, 16% glycerol, 0.8 mg/mL BSA.

- **Oligos**:

P5 AATGATACGGCGACCACCGA (20mer) &

P7 CAAGCAGAAGACGGCATACGA (21mer).

- **Dual Labelled Probe** (DLP) for TaqMan strategy only. Note: light sensitive!

Oligo modified with fluorochromes conjugated at either end 5’ reporter [6FAM] & 3’ quencher [TAMRA]: shRNAseq CCCTTGAATTCCGAGGCAGTA (21mer)

NOTE: Hybridisation probe-type depends on library origin, here shRNAseq. DLP aligns to sequence within adapters-harbouring amplicons.

- Optical 96 well plates P/N N801-0560 & thermo-resistant lids P/N 4313663, both from ABI.

- 100 nM Solexa standard (fully characterised sample) of equivalent size (bp) to samples to be determined (shRNAseq 117 bp, DGE 85 bp or RNAseq 200 bp).

- Gel purified samples, usual concentration range: 2-200 ng/uL, quantified at this stage by nanodrop & agilent chip.

# **RECIPE**

1. **Sybr-Green:**

**1X = 25 uL**

200 nM P5 primer…………...0.5 uL, 10 uM

200 nM P7 primer…………...0.5 uL, 10 uM

2X PCR mix…………...……12.5 uL

H2O………………………….9.0 uL

**Template…………………….2.5 uL, » 10 pM**

1. **TaqMan:**

**1X = 25 uL**

300 nM P5 primer…………...0.75 uL, 10 uM

300 nM P7 primer…………...0.75 uL, 10 uM

250 nM DLP…….….……….0.625 uL, 10 uM

2X PCR mix…….…………...12.5 uL

H2O………………………….7.875 uL

**Template…………………….2.5 uL, » 10 pM**

**
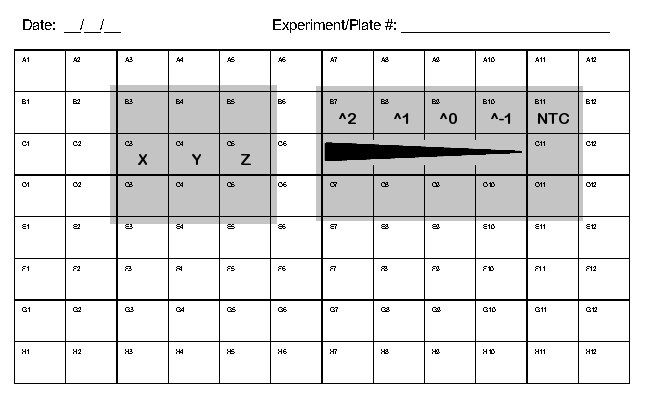
**

**LAYOUT example**

“**Standard” (not for “Fast” chemistry) thermal cycler protocol:**

- Stage 1: Initiation at 50 C for 2 min

- Stage 2: Hot start at 95 C for 10 min

- Stage 3: 40 repeats of 95 C for 15 s and 60 C for 1 min.

- Stage 4 (if SybrGreen): 95 C for 15 s, 60 C for 15 s and 95 C for 15 s.

# **PROCEDURE**

1. Prepare **10X Solexa stock** at 100 nM (» 7.7 ng/uL for 117 bp), using initial theoretical concentration as defined by Agilent Bioanalyser DNA1000 chip.

2. Prepare **working dilution 10 nM** (1:10 stock dilution)

3. Make 10-fold dilution series to obtain A-C (see bellow) for “unknown” samples (use dilution “C” at 10 pM to be confirmed by Real Time PCR), and A-E for reference (use B-E to construct standard curve B>C>D>E>**nought**: 100, 10, 1, 0.1, 0.0 pM respectively).

Note: Smaller dilution intervals also suitable (e.g. 1:5 series 100, 50, 10, 5, 1, 0.5, 0.1 pM).

Prepare volume that suffices: triplicate sampling, pipetting error, and serial dilution (e.g. 7.5 uL + 1.5 uL + 1 uL = 10 uL bare minimum per sample).

A) 1 nM

B) 0.1 nM = 100 pM 1exp 2 (^2)

C) 0.01 nM = 10 pM 1exp 1 (^1)

D) 0.001 nM = 1 pM 1exp 0 (^0)

E) 0.0001 nM = 0.1 pM = 100 fM 1exp -1 (^-1)

F) Nought (**NTC**: no template control)

4. Prepare sample layout.

5. Dispense 22.5 uL of your complete PCR mixture (lacking template) into each well to be used.

6. Distribute samples accordingly into designated wells. Note: useful to have them arranged into 8-tube 0.2 mL strips or a section of a 96wp to take replicates from with multi-channel.

7. Enter details of your run (e.g. detectors 6FAM or SYBRgreen, sample type std or unknown, adjusted volume, etc.) on SDS· software template at the RT-PCR instrument, whilst pulsing your plate on a centrifuge. If SYBRgreen via Absolute quantitation is the chosen method, add dissociation step at the end of the run settings.


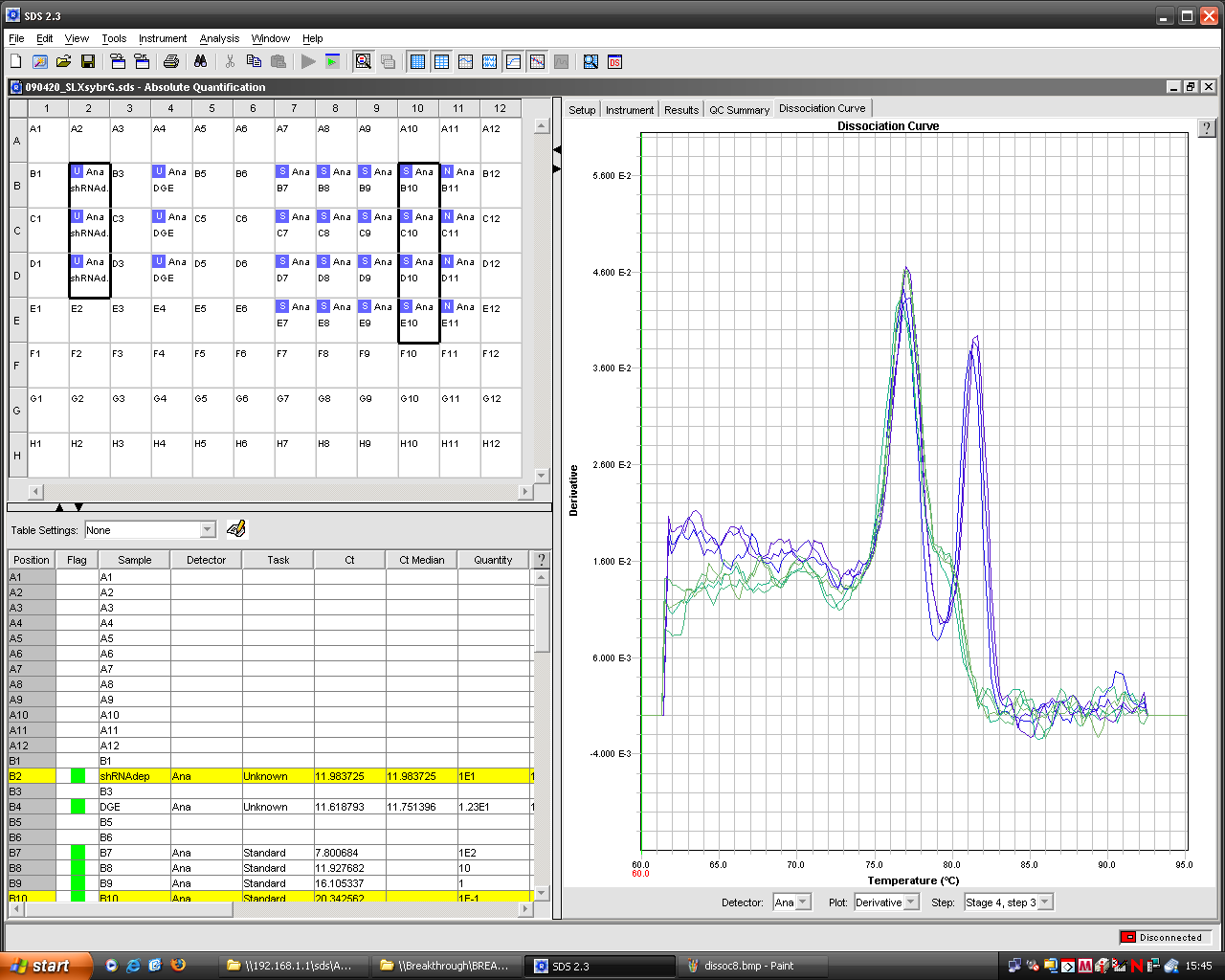


**PCR efficiency**

“Ef” = 10^(-1/slope)^-1

Optimal range:

3 < |slope std curve| < 4

PCR exponential nature

*N* = *N_0_* 2^n^

Post-amplification dissociation curve analysis: dimorphism of depleted set (due to shNS 16 % content or degradation)

EXAMPLE

# **SUMMARY**

**qPCR for Solexa samples**: A 10-fold dilution series of a characterised standard sample, at 100 nm, was used to obtain a standard curve ranging from 1: 10^3^ to a 1:10^6^-fold dilution of the standard. Real-time PCR were performed using universal PCR mix from Applied Biosystems in an ABI 7900 instrument. Triplicate measurements were carried out in 25 uL reaction, containing 10% template sample at a theoretical concentration of 10 pM as defined by Agilent Bioanalyser, P5 & P7 oligos. Solexa library concentrations were inferred by comparing measurements to the standard curve by SDS 2.2.1 software.

# **BASIC NOTIONS**

Fluorescence, given by release of hyb-probe when removed by amplifying polymerase or produced by SybrGreen dye when intercalated with dsDNA, is measured at the end of elongation phase in each cycle. The amount of DNA is inversely proportional to the amount of cycles required by the reporter signal to reach the detection threshold cycle (C_T_) of the plot, interception at which slope is optimal, exponential with maximum efficiency in amplification curves.

# **gDNA Issues**

Multiplex PCR as reporter of genomicDNA (template for shRNA libraries) integrity (van Beers *et al*., 2006). Note: Pure Gene kit (Qiagen) is column –free, thus shredding diminished. 4 pair of primers to amplify 4 differential products of GAPDH (chr12) differing in size (100-400 bp).

**
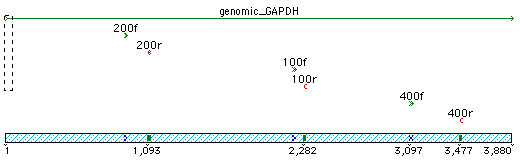
**

400F: ACAGTCCATGCCATCACTGC

400R: GCTTGACAAAGTGGTCGTTG

300F: AGGTGAGACATTCTTGCTGG


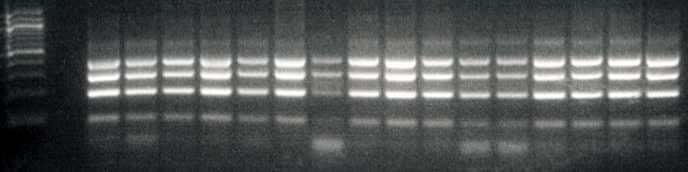
300R: TCCACTAACCAGTCAGCGTC

200F: AGGTGGAGCGAGGCTAGC

200R: TTTTGCGGTGGAAATGTCCT

100F: GTTCCAATATGATTCCACCC

100R: CTCCTGGAAGATGGTGATGG

Samples with different Solexa run outcome analysed retrospectively to test band/fragment display as prognostics for solexa quality performance: weak correlation, not predictor of success/failure under the protocol currently in use.

# **OTHER Estimations of Quality**

1) **NanoDrop** (Optical Density “OD” or Absorbance): Flags atypical spectral patterns. Purity assessment based on accepted nm ratios (i) 260:280 = 1.8; (ii) 260:230 = 2.0-2.2. Otherwise indication of contaminant (proteins, salt, etc).


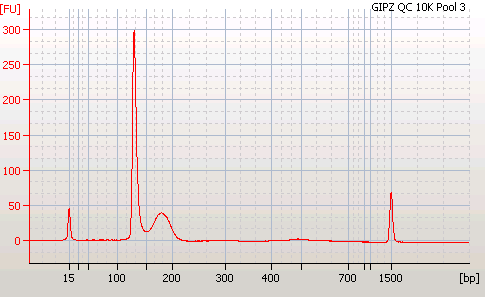
2) **Agilent Bioanalyser**: capillary electrophoresis renders sizing (bp) and amount (ng/uL & nM) from electropherogram.

3) **PicoGreen**: Assay employs dye that preferentially binds to dsDNA, and when so fluoresces. Fluorescence emission at fluorescein wavelength (535 nm) occurs upon excitation at 485 nm using a microplate reader. Quant-iT kit (Molecular Probes; invitrogen P-11496).

- Prepare fresh aqueous working 2x solution of PicoGreen reagent (susceptible to photo-degradation) by making a 200-fold dilution in TE of the concentrated DMSO stock.

- For 200 uL reaction/well, mix 100 uL test sample (1:100 in TE) with 100 uL 2x PicoG.


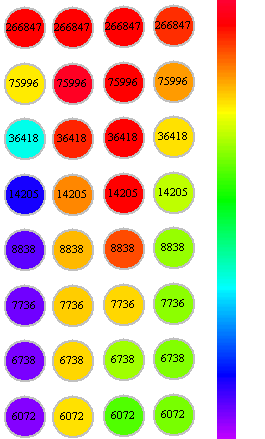
- Lambda DNA standard curve: dilute the provided stock 100 ug/mL 50-fold to make a 2 ug/mL stock solution further diluted 1:10 to 0.2 ug/mL (100 ng/uL if 200λ total volume).

**ng/uL**

100

25

10

2.5

1.0

0.5

0.25

0

Visual Scales

# 5. Solexa Run

Follow manufacturer’s guidelines, with the following important modification:

Use an alternative Solexa sequencing primer

mir30EcoR1Seqprimer: **TAGCCCCTTGAATTCCGAGGCAGTAGGCA**
